# Validation of the CoVID-TE model as a tool to predict thrombosis, bleeding, and mortality in the oncology patient with Sars-Cov-2 infection: a study by the SEOM cancer and thrombosis group

**DOI:** 10.1007/s12094-023-03233-2

**Published:** 2023-06-10

**Authors:** Manuel Sánchez Cánovas, David Fernández Garay, Francisco Gómez Martínez, Elena Brozos Vázquez, Miriam Lobo de Mena, Silvia García Adrián, Vilma Pacheco-Barcía, Diego Cacho Lavin, Eva Martínez de Castro, Ana Manuela Martín Fernández de Soignie, Elia Martínez, Ana Belén Rúperez Blanco, Ignacio García Escobar, Carmen Salvador Coloma, Noel Blaya Boluda, María Esperanza Guirao García, Mariano Gambín Arroniz, Andrés J. Muñoz Martín

**Affiliations:** 1 SEOM Cancer and Thrombosis Research Group (SEOM), Madrid, Spain; 2 Hematology and Medical Oncology Service, Hospital Universitario José María Morales Meseguer, Murcia, Spain; 3 Medical Oncology Service, Complejo Hospital Costa del Sol, Marbella, Spain; 4 Medical Oncology Service, Complejo Hospitalario Universitario de Santiago, Santiago de Compostela, Spain; 5 Medical Oncology Service, Hospital General Universitario de Valencia, Valencia, Spain; 6 Medical Oncology Service, Hospital Universitario de Móstoles, Madrid, Spain; 7 Medical Oncology Service, Hospital Universitario de Torrejón, Madrid, Spain; 8 Medical Oncology Service, Hospital Universitario Marqués de Valdecilla, Instituto de Investigación IDIVAL, Santander, Spain; 9 Medical Oncology Service, Hospital de Fuenlabrada, Madrid, Spain; 10 Medical Oncology Service, Hospital Universitario de Toledo, Toledo, Spain; 11 Medical Oncology Service, Hospital Lluis Alcanyis de Xativa, Valencia, Spain; 12 Internal Medicine Service, Hospital Universitario Reina Sofía, Murcia, Spain; 13 Medical Oncology Service, Hospital General Universitario Gregorio Marañón, Universidad Complutense, Madrid, Spain

**Keywords:** Sars-Cov-2, CoVID-TE score, Cancer, Thrombosis

## Abstract

**Purpose:**

The CoVID-TE model was developed with the aim of predicting venous thrombotic events (VTE) in cancer patients with Sars-Cov-2 infection. Moreover, it was capable of predicting hemorrhage and mortality 30 days following infection diagnosis. The model is pending validation.

**Methods/patients:**

Multicenter retrospective study (10 centers). Adult patients with active oncologic disease/ antineoplastic therapy with Sars-Cov-2 infection hospitalized between March 1, 2020 and March 1. 2022 were recruited. The primary endpoint was to study the association between the risk categories of the CoVID-TE model and the occurrence of thrombosis using the Chi-Square test. Secondary endpoints were to demonstrate the association between these categories and the occurrence of post-diagnostic Sars-Cov-2 bleeding/ death events. The Kaplan–Meier method was also used to compare mortality by stratification.

**Results:**

263 patients were enrolled. 59.3% were men with a median age of 67 years. 73.8% had stage IV disease and lung cancer was the most prevalent tumor (24%). A total of 86.7% had an ECOG 0–2 and 77.9% were receiving active antineoplastic therapy. After a median follow-up of 6.83 months, the incidence of VTE, bleeding, and death 90 days after Sars-Cov-2 diagnosis in the low-risk group was 3.9% (95% CI 1.9–7.9), 4.5% (95% CI 2.3–8.6), and 52.5% (95% CI 45.2–59.7), respectively. For the high-risk group it was 6% (95% CI 2.6–13.2), 9.6% (95% CI 5.0–17.9), and 58.0% (95% CI 45.3–66.1). The Chi-square test for trends detected no statistically significant association between these variables (p > 0.05). Median survival in the low-risk group was 10.15 months (95% CI 3.84–16.46), while in the high-risk group it was 3.68 months (95% CI 0.0–7.79). The differences detected were not statistically significant (p = 0.375).

**Conclusions:**

The data from our series does not validate of the CoVID-TE as a model to predict thrombosis, hemorrhage, or mortality in cancer patients with Sars-Cov-2 infection.

## Introduction

Venous thromboembolic disease (VTE), Sars-Cov-2 infection, and cancer are three variables seen as interrelated [1–3]. VTE increases mortality two to sixfold in cancer patients [4, 5].

In addition to this long-known correlation, Sars-Cov-2 infection in the cancer patient has been shown to have a mortality of 5.6% versus 2.3% cancer-free individuals [6, 7].

Finally, as for the association between Sars-Cov-2 infection and VTE, mortality among infected patients who develop thrombosis is 23% compared to 13%, among those in whom this complication does not occur [8].

These data were reason enough to attempt to identify which oncologic patient profile with Sars-Cov-2 has a higher risk of developing VTE and, therefore, should be prescribed prophylaxis and the dosage of said preventive therapy.

The CoVID-TE model [9] was designed with the aim of answering the questions previously posed. It is a model created on the basis of the “COVID-19 and Cancer Consortium registry (CCC19)” patient data. This model uses the following variables: cancer subtype (taking the Khorana model as a reference), history of VTE, need for ICU evaluation on admission, D-dimer elevation, antineoplastic therapy in the three months prior to Sars-Cov-2 infection, and ethnicity.

Based on the score obtained, subjects are classified as low risk for VTE (4.1% rate in the follow-up period) or high risk for VTE (11.3% rate in the follow-up period). Moreover, this model appeared to be capable of predicting hemorrhagic events as well as mortality in the first 30 days post-diagnosis of Sars-Cov-2 in the oncology patient.

However, the model is pending external validation. The Cancer and Thrombosis Group of the Spanish Society of Medical Oncology (SEOM) launched this research project to try to validate the model.

## Material and methods

A retrospective, multicenter study (involving 10 hospitals in Spain) was undertaken. Patients over 18 years of age with active oncologic disease or active antineoplastic therapy who had developed a Sars-Cov-2 infection requiring hospitalization were recruited. The recruitment period was between March 1, 2020 and March 1, 2022. A minimum follow-up of 90 days was required, unless this was impossible because of patient demise. Using the statistical software “Statulator beta” the necessary sample size was determined, estimating 260 patients [10].

The primary objective of the study was to validate the CoVID-TE model [9]. This model included the following variables: cancer subtype by original Khorana score (one point if high and very‐high risk categories based on the original Khorana score: pancreas, stomach, esophageal, lung, ovarian, kidney, bladder, testicular, lymphoma), history of VTE (two points if previous VTE), need for ICU evaluation on admission (two points if ICU triage on admission), D-dimer elevation (one point if high levels. The authors of the original article referred that “specific cut‐off could not be determined”), antineoplastic therapy in the three months prior to Sars-Cov-2 infection (one point if the patient is on treatment), and ethnicity (one point if the patient is non-Hispanic).

The association between the variables “development of thrombosis in the first 90 days following Sars-Cov-2 diagnosis” and “stratification as per the CoVID-TE model” was assessed using the Chi-Square test for trends. The test evaluated the null hypothesis that a higher CoVID-TE model category does not associate with an increased incidence of thrombosis. In all cases, the significance level α was 5%, accompanying 95% confidence interval (CI) ratios.

As for the secondary objectives, we studied the association between the variables “development of bleeding in the first 90 days post-diagnosis of Sars-Cov-2” and “death in the first 90 days post-diagnosis of Sars-Cov-2” with the variable “stratification according to the CoVID-TE model” following the same methodological basis as for the primary objective.

The Kaplan–Meier method was also used to compare mortality according to CoVID-TE model stratification. The log-rank test was used to establish whether the differences detected were statistically significant (p < 0.05). The statistical package utilized was SPSS 25.0 (IBM Corporation, Armonk, NY, USA).

Prior to its implementation, the study was submitted to the Ethics Committee of each participating center and obtained the corresponding approval. The processing, communication, and transfer of all personal data complied with the provisions of Organic Law 15/1999, dated December 13, 1999, regarding the protection of personal data and of Organic Law 3/2018, dated December 5, 2018, since its entry into force.

## Results

A total of 263 patients were recruited. The baseline characteristics of the sample are detailed in Table 1. Most patients included were male (59.3%, n = 156) with a median age of 67 years (interquartile range 59–76). The 73.8% (n = 194) of the study sample had stage IV disease at the time of Sars-Cov-2 infection diagnosis; the most prevalent tumors were lung, colorectal, and breast cancer with percentages of 24% (n = 63), 18.3% (n = 48), and 9.9% (n = 26), respectively. The predominant histology was adenocarcinoma (60.5%, n = 159).

**Table 1:** Baseline characteristics

Parameter	Subparameter	n = 263
Gender	Male	59.3% (n = 156)
	Female	40.7% (n = 107)
Smoking status	Never smoked	31.2% (n = 82)
	Active smoker	13.3% (n = 35)
	Ex-smoker	34.2% (n = 90)
	Unknown	21.3% (n = 56)
Medical history unrelated to the current cancer	HBP	57.8% (n = 152)
	DM	24.3% (n = 64)
	DLP	46.8% (n = 123)
	Chronic CV disease	20.2% (n = 53)
	COPD	19.4% (n = 51)
	Liver disease	7.2% (n = 19)
	CKD	9.88% (n = 26)
	Thrombophilia	0.4% (n = 1)
	Major surgery in previous 30 days	6.1% (n = 16)
Active anticoagulant therapy	No	79.1% (n = 208)
	Yes, at prophylaxis doses	14.8% (n = 39)
	Yes, at full doses	6.1% (n = 16)
Tumor stage	Stage I-III	26.2% (n = 69)
	Stage IV	73.8% (n = 194)
Primary tumor	Lung	24% (n = 63)
	Colorectal	18.3% (n = 48)
	Breast	9.9% (n = 26)
	Prostate	7.6% (n = 20)
	Pancreas	4.9% (n = 13)
	Gastric	4.6% (n = 12)
	Other	30.7% (n = 81)
Histology	Adenocarcinoma	60.5% (n = 159)
	Epidermoid	10.6% (n = 28)
	Microcytic/High Grade Neuroendocrine	5.7% (n = 15)
	Sarcoma/Stromal Tumor	4.2% (n = 11)
	Other	19% (n = 50)
Treatment modality	Not started	16% (n = 42)
	Adjuvant	11% (n = 29)
	Neoadjuvant	12.2% (n = 32)
	First-line metastatic disease	29.3% (n = 77)
	Second-line metastatic disease	14.1% (n = 37)
	Third or subsequent line of metastatic disease	11.4% (n = 30)
	Palliative care	6.1% (n = 16)
Treatment regimen	No treatment	22% (n = 58)
	Chemotherapy	41.8% (n = 110)
	Hormone therapy	3.3% (n = 7)
	Targeted molecular therapy	4.3% (n = 9)
	Immunotherapy	8.4% (n = 22)
	Radiotherapy	2.3% (n = 6)
	Surgery	0.8% (n = 2)
	Chemotherapy + Immunotherapy	0.8% (n = 2)
	Chemotherapy + Targeted molecular therapy	5.3% (n = 14)
	Chemotherapy + Radiotherapy	2.3% (n = 6)
	Hormone therapy + Targeted molecular therapy	2.3% (n = 6)
	Targeted molecular therapy + Immunotherapy	0.4% (n = 1)
ECOG	0	16.7% (n = 44)
	1	46.8% (n = 123)
	2	23.2% (n = 61)
	3	11% (n = 29)
	4	2.3% (n = 6)

*CKD* chronic kidney disease, *COPD* chronic obstructive pulmonary disease, *CV* cardiovascular disease, *CVD* cerebrovascular disease, *DLP* dyslipemia, *DM* diabetes mellitus, *HBP* high blood pressure, *VTE* venous thromboembolism

Approximately 77.9% (n = 206) of the participants were receiving some form of antineoplastic treatment (adjuvant, neoadjuvant, first or successive lines for metastatic disease). The type of treatment most commonly prescribed was monochemotherapy (41.8%, n = 110). Most of the sample (63.5%, n = 167) had a good functional status, with an ECOG of 0 or 1.

As for the items that comprise the CoVID-TE model, their prevalence is reflected in Table 2. The most of the sample (67.68%, n = 178) was stratified as low risk. On the other hand approximately a third of the sample (32.32%, n = 85) was stratified as high risk.

**Table 2:** CoVID-TE items

Parameter	n = 263
Cancer subtype by original Khorana score^1^	40.3% (n = 106)
VTE history (lifetime)	9.1% (n = 24)
ICU triage on admission	8.4% (n = 22)
Elevated D‐dimer^2^	83.3% (n = 219)
Therapy (recent systemic last 3 months)	68.4% (n = 180)
Non‐Hispanic ethnicity	1.1% (n = 3)

^1^Combined high and very‐high risk categories based on the original Khorana score: pancreas, stomach, esophageal, lung, ovarian, kidney, bladder, testicular, lymphoma. ^2^Taking in consideration that in the original article [9] there was not a specific cut-off, each participant center used its own laboratory values to establish when was the d-dimer elevated

*ICU* intensive care unit, *VTE* venous thromboembolism

After a median follow-up of 6.83 months, the incidence of VTE 90 days following diagnosis of Sars-Cov-2 in the low-risk group was 3.9% (95% CI 1.9–7.9) while in the high-risk group it was 6% (95% CI 2.6–13.2). The Chi-Square test for trends failed to reveal a statistically significant association between the two variables (p = 0.459).

In regards to bleeding events, the incidence of such events 90 days after Sars-Cov-2 diagnosis in the low-risk group was 4.5% (95% CI 2.3–8.6) whereas in the high-risk group, it was 9.6% (95% CI 5.0–17.9). Again, the Chi-Square test for trends did not result in a statistically significant association between the two variables (p = 0.104).

Finally, concerning the 90-day mortality rate following Sars-Cov-2 diagnosis, the percentage of patients who died in the low-risk group was 52.5% (95% CI 45.2–59.7) compared to 58.0% (95% CI 45.3–66.1) in the high-risk group. Similarly, after applying the Chi-Square test for trends, no statistically significant correlation was established between the two variables (p = 0.602).

**Fig. 1:**
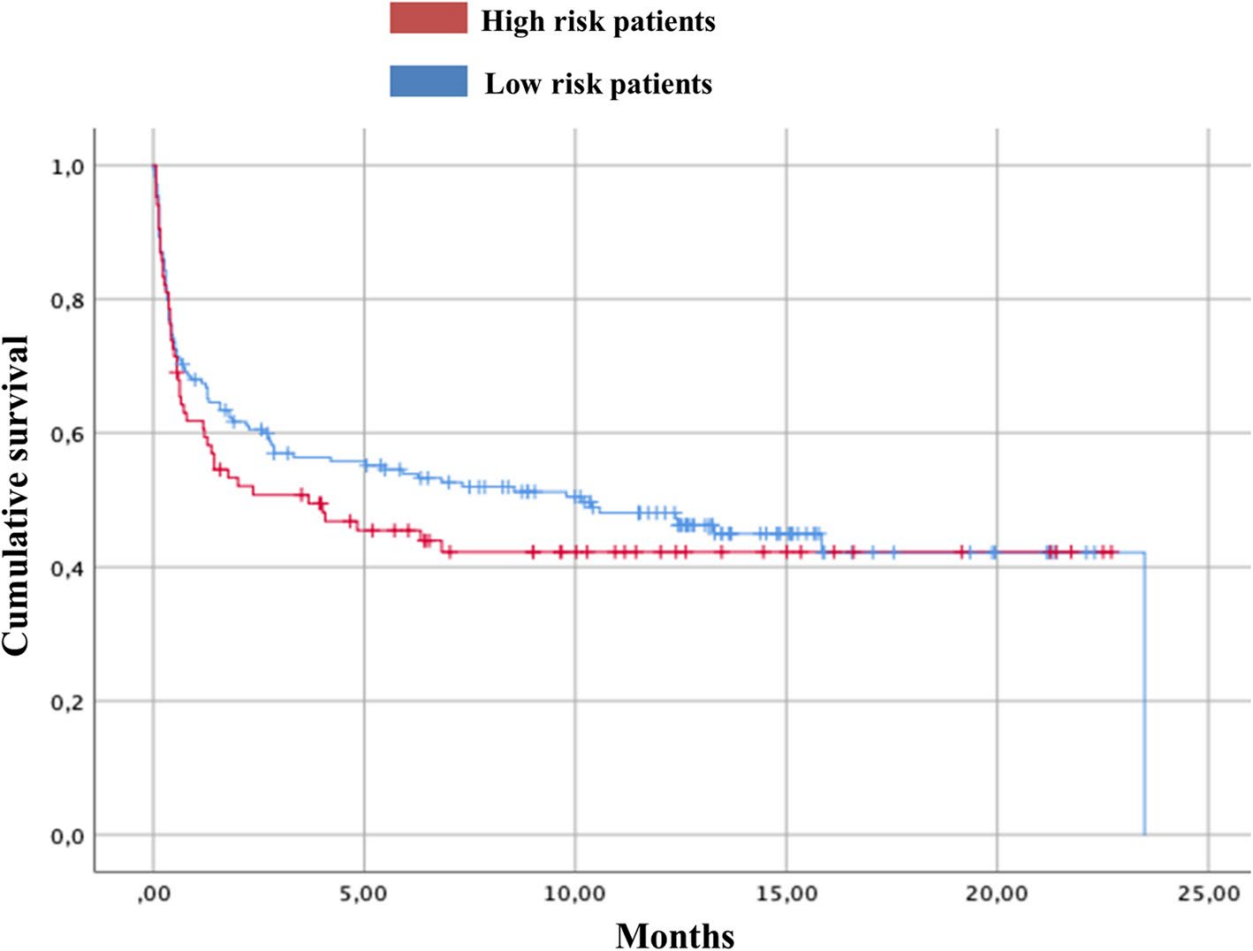
Survival analysis Kaplan Meier curve comparing overall survival (since initation Sars-Cov-2 diagnosis) of high risk–CoVID-TE patients *versus* low risk–CoVID-TE patients

The survival analysis (Fig. 1) reflected that the median survival in the low-risk group was 10.15 months (95% CI 3.84–16.46) while in the high-risk group it was 3.68 months (95% CI 0.0–7.79). The differences detected were not statistically significant (p = 0.375).

## Discussion

The rapid progress of the Sars-Cov-2 pandemic, as well as its severity, has prompted a tremendous research effort to better understand and manage both the disease and its complications. VTE represents one of such complications and medical debate forum has attempted to elucidate when VTE prophylaxis should be indicated and whether anticoagulant therapy at intermediate or even full doses should be considered upfront.

In cancer patients, VTE prophylaxis in hospitalized patients is fully implemented and widespread. The leading clinical practice guidelines recommend prescribing it in any circumstance of hospitalization, with the exception of individuals in their last days of life or young people admitted to receive antineoplastic therapy [11–15].

In a context of Sars-Cov-2 infection in the hospitalized cancer patient, the reasonable doubt is whether anticoagulation should be undertaken at intermediate doses (versus prophylactic doses). In this scenario, the availability of a model capable of identifying patients at high risk for VTE would aid in decision making regarding the approach to anticoagulation therapy.

Developing the CoVID-TE model [9] was a first step toward overcoming these issues. Our group believed it was necessary to conduct an external validation that might promote its dissemination and use. The data from our series indicated that subjects classified as high-risk (vs. low-risk) had a higher incidence of thrombosis (6% vs. 3.9%), bleeding (9.6% vs. 4.5%), and mortality (56% vs. 52.5%) in the first 30 days after Sars-Cov-2 diagnosis. However, none of these differences resulted statistically significant; thus, we have been unable to confirm the validation of this model.

We have endeavored to identify the reasons why we have not managed to validate it. If we compare the original series [9] with ours, certain differences emerge as being worthy of note. The first of these is the smaller sample size (263 vs 2457 patients), although we have adapted to the sample size we calculated as necessary to validate the model.

If we examine the prevalence of the various factors that make up the model in both series, the only point that can be viewed as similar in both is the history of VTE. This item was positive in 11% of the participants in the original series [9], as opposed to 9.1% in our sample.

The initial assessment performed in the ICU, together with a history of VTE, represents the variable that carries the most weight in the CoVID-TE index. In our series, only 8.4% were evaluated by the ICU upon admission, whereas in the initial cohort [9], 16% were admitted by this service. This datum may be synonymous with greater severity in the initial study population, although it may also be indicative that, from an oncologic point of view, the population in our study has more characteristics that might lessen the likelihood of ICU admission.

Such characteristics include tumor staging. In our series, 73.8% of the patients had stage IV disease. The original series [9] only reports 29% of their sample as having distant disease. This confers a worse prognosis on the population recruited by our research group. It should also be noted that the original series [9] includes 24% of the cases with hematologic malignancies. The prognosis for these disorders differs from that of solid tumors, which may affect the results obtained. One of the main strengths of our study is that it consists solely of patients with solid tumors and, to our knowledge, it is the first series of cases in which validation of the CoVID-TE model has been attempted.

Returning to the variables that constitute the CoVID-TE model, the existing differences in the prevalence of high-risk tumors according to the Khorana scale are also worth highlighting. The initial series [9] reports 26% high-risk tumors, while our population includes 68.1% of such lesions. Concerning the prevalence of tumor pathology in both series, most of our series had lung cancer (24% vs. 9% in the initial series), whereas the most prevalent tumor in the original article was prostate (15% vs. 7.6% in our series). Prostate cancer has a better prognosis and less thrombotic risk than lung cancer.

Likewise, it should be stressed that our series has a higher prevalence of tumors having a worse prognosis and more commonly associated with thrombosis (both based on molecular biology and on the type of treatment administered) compared to the initial series [9]: we enrolled 18.3% of patients with colorectal cancer (as opposed to 8% in the initial study), 4.9% with pancreatic cancer (in contrast to 2% in the original series), and 4.6% with esophago-gastric cancer (versus 4% in the first series).

Continuing with the CoVID-TE model variables, our series included a higher proportion of individuals on active treatment (68.4% vs. 36% in the initial series), more subjects with elevated D-dimer (83.3% vs. 49.9% in the initial series), as well as pronounced differences in the percentage of non-Hispanic patients (1.1% vs. 77% in the original study population).

In the first 90 days post-diagnosis of Sars-Cov-2 infection, the incidence of VTE and hemorrhage in the high-risk group was 6% and 9.6%, respectively, compared to 11.3% and 10% in the initial series [9]. The bleeding data yielded similar results. Nevertheless, the higher incidence of thrombosis in the original series is particularly compelling bearing in mind that, a priori, there are more oncologic prothrombotic risk factors in our cohort. This may not be due entirely to these factors. It is likely that epidemiological variables, non-oncologic comorbidities, and even the most prevalent variant of Sars-Cov-2 at the time the study was conducted may have contributed to these differences and, therefore, to the fact that we were unable to validate the scale in our cohort.

Prior to the discussion of the mortality data, we aimed to justify why we analyzed the variable of death 90 days after diagnosis of Sars-Cov-2 and not at 30 days as proposed by the initial model. Thrombosis/ hemorrhagic events can account for mortality in the cancer patient. Let us assume that a Sars-Cov-2 infected oncology patient develops thrombosis within 30 days post-infection. It is conceivable that such a patient could die in the following weeks from a recurrence of VTE or even from a hemorrhagic event related to anticoagulant therapy.

Furthermore, the fact that hospital stay may be prolonged for more than 30 days in this type of patient or the effect that Sars-Cov-2 infection may have on interruptions (transitory or definitive) in antineoplastic therapy is not insignificant. This can have a deleterious effect on the person in terms of oncologic prognosis.

In our series, at 90 days, there was a similar percentage of death among cancer patients, whether they were stratified as low risk (52.5%) or high risk (56%), albeit no statistically significant correlation was established. Consequently, and based on the data from our series, it appears that this scale may be weaker for performing mortality analyses. This is not the case for thrombosis and bleeding events, in which the prevalence of these complications is approximately 1.5 to 2.5 times higher in the high-risk category (6% and 9.6%, respectively) than in the low-risk category (3.9% and 4.5%). Even so, the fact that the chi-square test failed to yield statistically significant results must be underscored, thereby precluding the validation of the scale with our study population.

Our study has some limitations. The retrospective nature of the study could engage the results observed. The development and deployment of anti-Sars-Cov-2 vaccines and antiviral therapies, such as nirmatrelvir-ritonavir, have led to a substantial decrease in the percentage of patients who develop severe infection and require hospitalization [16]. This means that if a prospective study was designed, recruitment would be slower and have much less of a clinical impact than in the initial phases of the pandemic.

In the design phase of the study, we calculated that we would need a total of 260 patients. However, in the initial article [9] they analyzed a total of 2804 patients. Therefore, a limitation of our study may be the low sample size compared to the original work.

Another limitation to keep in mind is that, as far as we know, this is the first series of cases in which validation of the CoVID-TE model has been attempted. As we are not aware of other external validations, we cannot compare our data with any series other than the original one used to design the model. Hence, it is possible that the same results will not be reached with other cohorts.

## Conclusions

The data from our series do not endorse validation of the CoVID-TE model to predict thrombosis, bleeding, or mortality in the oncologic patient with Sars-Cov-2 infection.

## Data Availability

The authors declare the availability of data analyzed in this study.
